# Strong restructuration of skin microbiota during captivity challenges *ex-situ* conservation of amphibians

**DOI:** 10.3389/fmicb.2023.1111018

**Published:** 2023-02-20

**Authors:** Léa Fieschi-Méric, Pauline Van Leeuwen, Kevin Hopkins, Marie Bournonville, Mathieu Denoël, David Lesbarrères

**Affiliations:** ^1^Laboratory of Ecology and Conservation of Amphibians (LECA), Freshwater and OCeanic science Unit of reSearch (FOCUS), Université de Liège, Liège, Belgium; ^2^Biology Department, Laurentian University, Sudbury, ON, Canada; ^3^Institute of Zoology, Zoological Society of London (ZSL), London, United Kingdom; ^4^Aquarium-Muséum de l’Université de Liège, Freshwater and OCeanic science Unit of reSearch (FOCUS), Liège, Belgium; ^5^Environment and Climate Change Canada, National Wildlife Research Centre, Ottawa, ON, Canada

**Keywords:** *Batrachochytrium dendrobatidis*, seasonal habitat-shifts, holobiont conservation, microbiota flexibility, population translocation, overwintering, survival assurance populations

## Abstract

In response to the current worldwide amphibian extinction crisis, conservation instances have encouraged the establishment of *ex-situ* collections for endangered species. The resulting assurance populations are managed under strict biosecure protocols, often involving artificial cycles of temperature and humidity to induce active and overwintering phases, which likely affect the bacterial symbionts living on the amphibian skin. However, the skin microbiota is an important first line of defense against pathogens that can cause amphibian declines, such as the chytrid *Batrachochytrium dendrobatidis (Bd)*. Determining whether current husbandry practices for assurance populations might deplete amphibians from their symbionts is therefore essential to conservation success. Here, we characterize the effect of the transitions from the wild to captivity, and between aquatic and overwintering phases, on the skin microbiota of two newt species. While our results confirm differential selectivity of skin microbiota between species, they underscore that captivity and phase-shifts similarly affect their community structure. More specifically, the translocation *ex-situ* is associated with rapid impoverishment, decrease in alpha diversity and strong species turnover of bacterial communities. Shifts between active and overwintering phases also cause changes in the diversity and composition of the microbiota, and on the prevalence of *Bd*-inhibitory phylotypes. Altogether, our results suggest that current husbandry practices strongly restructure the amphibian skin microbiota. Although it remains to be determined whether these changes are reversible or have deleterious effects on their hosts, we discuss methods to limit microbial diversity loss *ex-situ* and emphasize the importance of integrating bacterial communities to applied amphibian conservation.

## 1. Introduction

Amphibians constitute the most imperiled vertebrate class on earth, with over a third of species globally threatened with extinction, mainly because of habitat loss, invasive species, infectious diseases, and pollution (Blaustein et al., 2011; IUCN, 2022). In view of their accelerating rate of extinction (McCallum, 2007), the International Union for the Conservation of Nature (IUCN) published the first Amphibian Conservation Action Plan (ACAP) in 2007 (Gascon, 2007). The ACAP recommended the establishment of *ex-situ* survival assurance populations to safeguard those species most at risk and stock for potential future reintroduction programs (Mendelson et al., 2007). Such collections typically involve more intensive management than display zoo populations (Mendelson, 2018), with increased biosecurity procedures (Pessier and Mendelson, 2010; Jensen et al., 2021) and sometimes limited co-housing of individuals (Gray et al., 2018) to reduce risks of pathogen spread, as well as artificial cycles of temperature and humidity to reflect natural life conditions and maximize reproduction outputs (Santana et al., 2015; Calatayud et al., 2021; Silla et al., 2021). Following the publication of the ACAP, over 800 species were classified as *ex-situ* rescue or research priority species (Dawson et al., 2016), and 77 captive breeding programs were created in the space of 7 years—of which 43% comprised rescues to establish survival assurance populations (Harding et al., 2016).

Although these *ex-situ* collections have considerably contributed to the survival of many species (Griffiths and Pavajeau, 2008; Tapley et al., 2015), their conservation efforts focus on amphibian hosts only, with little consideration for their microbial symbionts (Trevelline et al., 2019). However, increasing evidence shows the importance of bacterial communities, referred to as “microbiota,” for the health of their host (Douglas, 2018; Peixoto et al., 2021). More specifically, the skin microbiota of amphibians plays a crucial role against the deadly chytridiomycosis (Rebollar et al., 2020)—a skin disease notably transmitted by the chytrid fungus *Batrachochytrium dendrobatidis (Bd)*, already responsible for the decline of many amphibian species, including several presumed extinctions (Scheele et al., 2019). A few bacteria with inhibitory activity against this pathogen have been identified in the natural skin microbiota of amphibians resisting infection (Brucker et al., 2008; Woodhams et al., 2018). Interestingly, not all amphibians possess such protective symbionts, and their susceptibility to the disease is principally explained by the composition of their skin bacterial communities (Piovia-Scott et al., 2017; Rebollar et al., 2020). Moreover, amphibian skin microbiota are dynamic; in the wild, their structure naturally changes throughout seasons, although the temporal variation of protective symbionts is less clear (Bletz et al., 2017). Considering that the amphibian skin microbiota is largely assembled from bacteria present in the environment (Walke et al., 2014; Bird et al., 2018), it is likely restructured when wild amphibians are moved to captivity, as a consequence of the drastic reduction of environmental reservoirs of bacteria *ex-situ*.

Several studies comparing wild and captive amphibians have confirmed differences in composition and diversity of their skin microbiota (Becker et al., 2014; Kueneman et al., 2016a; Sabino-Pinto et al., 2016), but only two monitored its reorganization throughout the transition from the wild to captivity (Loudon et al., 2014; Bates et al., 2019). These latter studies found that the skin microbiota of amphibians placed *ex-situ* for a few weeks significantly decreased in diversity and changed in community composition, but they were limited to a short period and did not analyze the consequences of captivity on multiple *Bd*-inhibitory taxa. Moreover, the effect of a transition from the wild to captivity under specific management protocols used for survival assurance populations (i.e., combining biosecurity protocols, restricted social groups, and cycles of active and overwintering phases) on the amphibian skin microbiota was never investigated to our knowledge. Yet, determining whether such *ex-situ* conservation approaches could have unintended deleterious impacts on the natural defenses of amphibians against pathogens should be a priority. Diverse and rich microbiota are generally associated with better health and stronger *Bd* inhibition in amphibians (Longo et al., 2015; Bates et al., 2018; Harrison et al., 2019); the potential restructuration of their skin bacterial assemblages could thus put them at higher risk of infection by emerging diseases if reintroduced into the wild.

**Figure 1:**
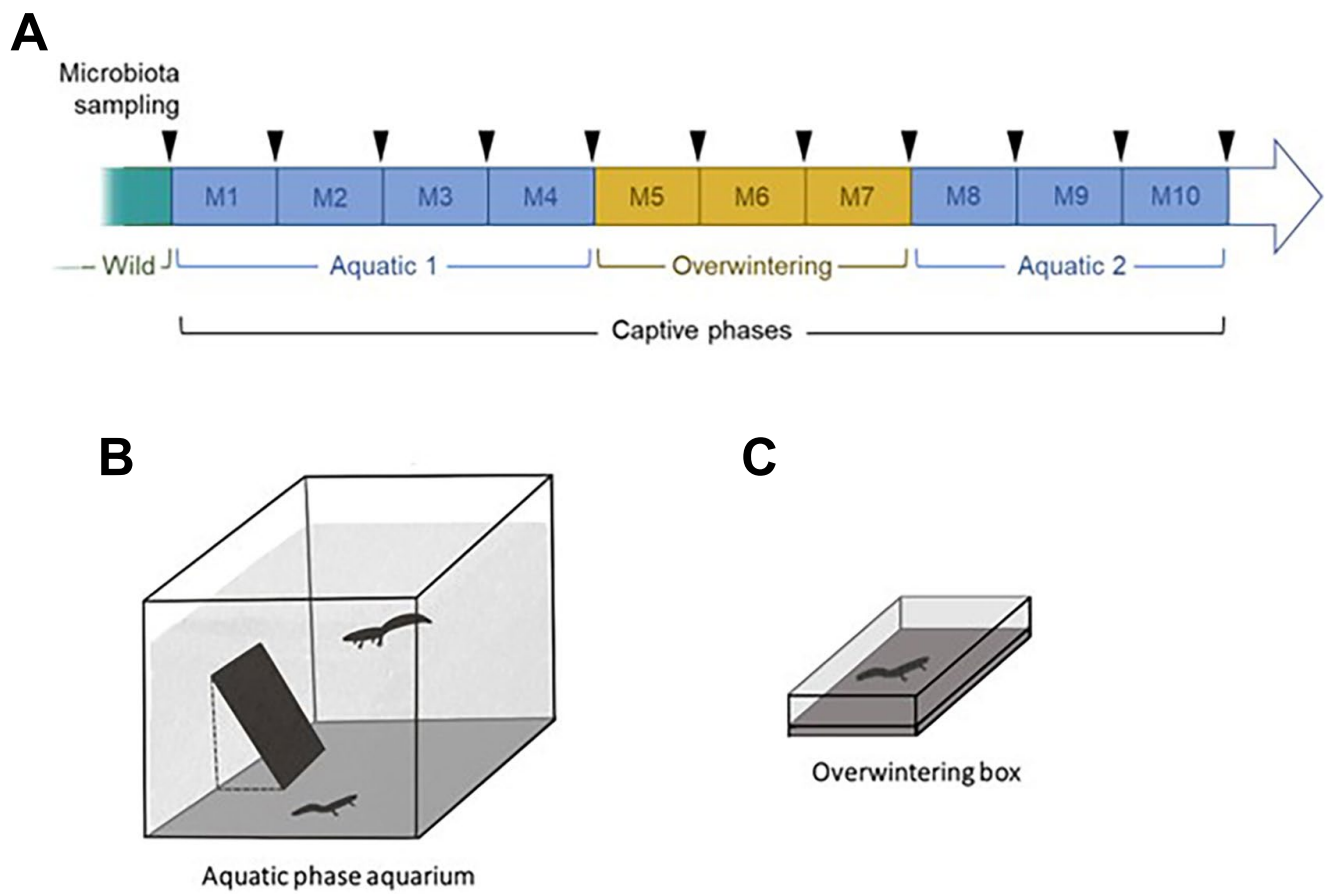
Schematic summary of the experimental plan used in this study **(A)**. Typical management protocols for survival assurance populations were used to maintain the collection of newts, combining biosecurity protocols, restricted social groups, and cycles of active and overwintering phases. Upon capture in the wild, 40 newts (20 per species) were placed by pairs in aquatic aquaria for 4 months without mixing species (Aquatic 1: M1 to M4; **B**). They were then transferred in individual containers containing wet cork and placed at 4°C to induce a terrestrial overwintering phase for 3 months (Overwintering: M5 to M7; **C**). The same pairs of newts as in the first aquatic phase were reunited in a second aquatic phase for 3 months (Aquatic 2: M8 to M10). The skin microbiota of the newts was sampled monthly (arrows).

We explored this gap in knowledge using two amphibian species commonly held in *ex-situ* institutions (data from ZIMS for Studbooks; Species 360, 2021): the alpine newt, *Ichthyosaura alpestris* and the palmate newt, *Lissotriton helveticus*. These species were also selected for their contrasting susceptibility to *Bd*; since this pathogen can be lethal to alpine newts but not to palmate newts (Cheatsazan et al., 2013; Miaud et al., 2016), we expected different proportions of *Bd*-inhibitory taxa among their natural microbiota. We captured wild adult newts and established an *ex-situ* collection managed using standard protocols for amphibian survival assurance populations, including biosecurity measures to reduce the risk of introducing exogenous micro-organisms (BSL-2 standards), and alternating cycles of active (aquatic) and overwintering (terrestrial) phases (Figure 1). Skin microbiota samples were collected monthly, and were characterized through high-throughput sequencing (Gołębiewski and Tretyn, 2020) to determine the evolution of bacterial communities and of *Bd*-inhibitory phylotypes through the transition from the wild to captivity, and throughout 10 months in captivity. We hypothesized that the two species of newts would have distinguishable skin bacterial communities in the wild, but that they would be similarly affected by their transfer into captivity. We predicted a reduction in alpha diversity caused by the limited diversity of exogenous bacteria *ex-situ*, and a major species turnover during the transitions between aquatic and terrestrial phases. Finally, we predicted that the relative abundance of *Bd*-inhibitory bacteria would decrease as a result of the relaxed selection pressure caused by biosecurity protocols.

## 2. Materials and methods

### 2.1. Establishment of the *ex-situ* collection of newts

We collected 40 metamorphosed adult newts (alpine newts, *Ichthyosaura alpestris*, ALP: 10 males and 10 females; palmate newts, *Lissotriton helveticus,* PAL: 10 males and 10 females) in the same pond in the Larzac plateau, France, on May 28, 2018. The pond is permanent, has a natural substrate, is highly vegetated and is surrounded by agricultural lands (crops) and a few trees. All animals were immediately transported to the laboratory in individual containers. Upon arrival in the animal housing facilities, the newts were randomly placed in intraspecific heterosexual pairs into 135 l aquaria (60 × 60 cm, 50 cm water depth; Figure 1A) filled with dechlorinated water (aerated for 48 h to evaporate chlorine) and oxygenated with air pumps. Photoperiod (14 h light, 325 lux) and temperature (mean ± SE = 16.5 ± 0.7°C) reflected environmental conditions from the capture location. Partial water changes, oxygen concentration measures and titration of reactive nitrogen forms were conducted regularly to keep the water quality constant. Each pair of newts was fed every 2 days at 18:00, with 400 mg of defrosted bloodworms (Ocean Nutrition, Dartmouth, Canada). The food was frozen to reduce chances of transmission of exogenous bacteria and zoonotic agents reported in live bloodworms (Rouf and Rigney, 1993; Broza and Halpern, 2001; Moore et al., 2003).

After 4 months of this first aquatic phase, the newts were placed in individual plastic containers (23 × 15 × 6 cm; Figure 1C) filled with a cork substratum and kept in a dark, refrigerated, incubator gradually set to 4°C to create a 3-months long terrestrial overwintering period. The newts were not fed in order to comply with their natural metabolism during this phase, but were sprayed 3 times with dechlorinated tap-water (30 cm distance from the newts) every 2 days to keep their skin moist. The incubator’s temperature was progressively increased before the start of the second aquatic phase, which was conducted in the exact same conditions as the first one, and lasted 3 months. The same pairs of individuals from the first aquatic phase were reunited (Figure 1B). Co-housing the newts during their aquatic phases enabled them to express mating behavior, which naturally occurs in the wild and is likely necessary to their welfare. Nevertheless, our study species can overwinter alone in the wild, so we separated them during the overwintering phase.

The equipment used for capture, transport and maintenance of the newts was thoroughly washed and disinfected before and after use with a 3% VIRKON solution (Van Rooij et al., 2017).

### 2.2. Microbiota sampling

The skin microbiota of the newts was sampled upon capture in the field, and monthly throughout captivity (11 sampling events per newt; Figure 1A). Samples collection consisted of a non-invasive skin swabbing. Each individual was held with a new pair of nitrile gloves and was gently rubbed with two sterile swabs (MW100 rayon tipped dry swab, MWE, Corsham, UK) as follows: 10 strikes back-and-forth on the ventrum, five on each side of the tail, five on each side of the back, five rolls on each hand and foot. The swabs were preserved dry, on ice upon collection in the field, at -25°C at the field station, and at-80°C after being transported to the laboratory, until further processing. One swab was used for the microbiota analysis and the other was kept for long-term archiving in our laboratory.

Despite our biosecurity measures, exogenous bacteria could be introduced through the water poured into the aquaria or through the cork used as substrate in the overwintering containers. To identify these environmental bacteria, swabs were monthly stirred 20 times in the water of control aquaria during aquatic phases, and rubbed 10 times on the cork of overwintering control containers during the terrestrial phase. These control aquaria (*n* = 3) and containers (*n* = 3) did not contain any newts but were maintained in the same conditions as the ones that did.

### 2.3. Microbiota sequencing and bioinformatics

DNA was extracted from the swabs using the DNeasy PowerSoil Pro kit (QIAGEN, Hilden, Germany), following the manufacturer’s instructions and including non-template controls (NTCs). Following the protocol outlined in Harrison et al. (2019), library preparation (using 515F and 806R primers) and community amplicon sequencing of the hypervariable V4 region of the 16S ribosomal RNA gene (~254 bp) were conducted on a MiSeq system (Illumina, San Diego, California, USA), at a depth of 30,000 reads. Demultiplexed sequences were processed using DADA2 v.1.8 (Callahan et al., 2016). Forward and reverse reads were truncated at decreasing quality (respectively 240 and 150 bp), and chimeric Amplicon Sequence Variants (ASVs) were removed by reconstruction against more abundant parent ASVs. Taxonomy was assigned to representative sequences using a naive Bayesian classifier implemented in QIIME 2, trained against EMPO 3 “animal surface” habitat-specific taxonomic weights (Kaehler et al., 2019). Assignments were accepted above a 0.7 confidence threshold. To identify symbiotic phylotypes with known inhibitory activity against *Bd*, representative sequences were aligned to the Antifungal Isolates Database (Woodhams et al., 2015) in QIIME 2.

Preprocessing of the sequences was carried out using the R package phyloseq (McMurdie and Holmes, 2013). Only bacterial sequences were kept, and 13 contaminant ASVs identified from NTCs were removed using the R package decontam (Davis et al., 2018). To address the uneven depth of coverage, all samples were normalized by rarefaction without replacement (Cameron et al., 2021) at 22140 reads. ASVs with no taxonomic affiliation at the phylum level, and spurious ASVs making up less than 0.005% of the total reads, were filtered out from the data (Bokulich et al., 2013). The final dataset was comprised of 12,255 ASVs, across a total of 436 newt samples.

### 2.4. Statistical analysis

Differences in alpha (within-sample diversity) and beta (among-samples dissimilarity) diversity between species and phases were investigated using statistical tests of similar structure (described below). Alpha diversity was quantified using Chao1 (estimated ASV richness) and Shannon (estimated ASV evenness) indices. Factors potentially influencing these indices were included in linear and linear mixed models (described below), and tested through analyses of variance (ANOVA). In cases where the residuals of the models did not meet the assumptions of normality and homoscedasticity associated with ANOVAs, a log-transformation of the response variable was successful at resolving these assumptions. Pairwise contrasts between phases were tested using estimated marginal means. Beta diversity was quantified using the weighted Unifrac distance (phylogenetic distance weighted by species abundance information) to investigate differences in community structure among samples (Lozupone et al., 2011). Permutational multivariate analyses of variance (PERMANOVAs) implemented using the adonis function (*n* = 9,999 permutations) were used to test similar models as for the alpha diversity. Pairwise differences between phases were tested using a pairwise adonis test. Differences in within-group variation in community structure (i.e., differences in compositional variance of microbiota) were investigated using betadisper tests. To identify bacterial taxa responsible for the observed differences in community structure among samples, differential abundance analyses were completed on unrarefied data (436 newt samples, 14,185 taxa) using DEseq2 (Love et al., 2014).

Initial differences in microbiota diversity between wild samples were investigated using a model that included species, sex, their interaction and individual snout-vent length (SVL) as fixed effects. For both alpha and beta diversity indices, this model showed that sex and SVL (mean ± SE = 4.19 ± 0.08 cm) had no effect on the diversity of the microbiota, therefore these variables were not included in subsequent models for parsimony purposes. A second model restricted on data from the two first sampling events (i.e., in the wild and after 1 month *ex-situ*) (Figure 1A) was built to investigate short-term changes in diversity over the transition from the wild to captivity. It included species, time of sampling, and their interaction as fixed effects, and individual identity as a random intercept. Lastly, the effect of phase-shifts on the microbiota was investigated using the full dataset, through a model including species, phase and their interaction as fixed effects. That model included individual identity, aquarium identity, as well as month of sampling as random effects. Estimates associated with the covariates in all models were deemed significant if associated with *p*-values below a 0.05 threshold. If interaction terms were not statistically significant, models were rebuilt without them (Beck and Bliwise, 2014).

All analyses were conducted in the R environment v.4.1.0 (R Core Team, 2022). Shared ASVs between species and phases were visualized using Venn diagrams created in the R package ggVennDiagram (Gao et al., 2021). Variation in beta diversity was visualized using Principal Coordinates Analyses (PCoA), built using the R package vegan (Oksanen et al., 2020). Other graphical representations were plotted using the R packages ggplot2 (Wickham, 2016) and ggpubr (Kassambara, 2019). All data and code are publicly available at Figshare repository.^1^

## 3. Results

### 3.1. Differences in microbiota structure between newt species in the wild

**Figure 2:**
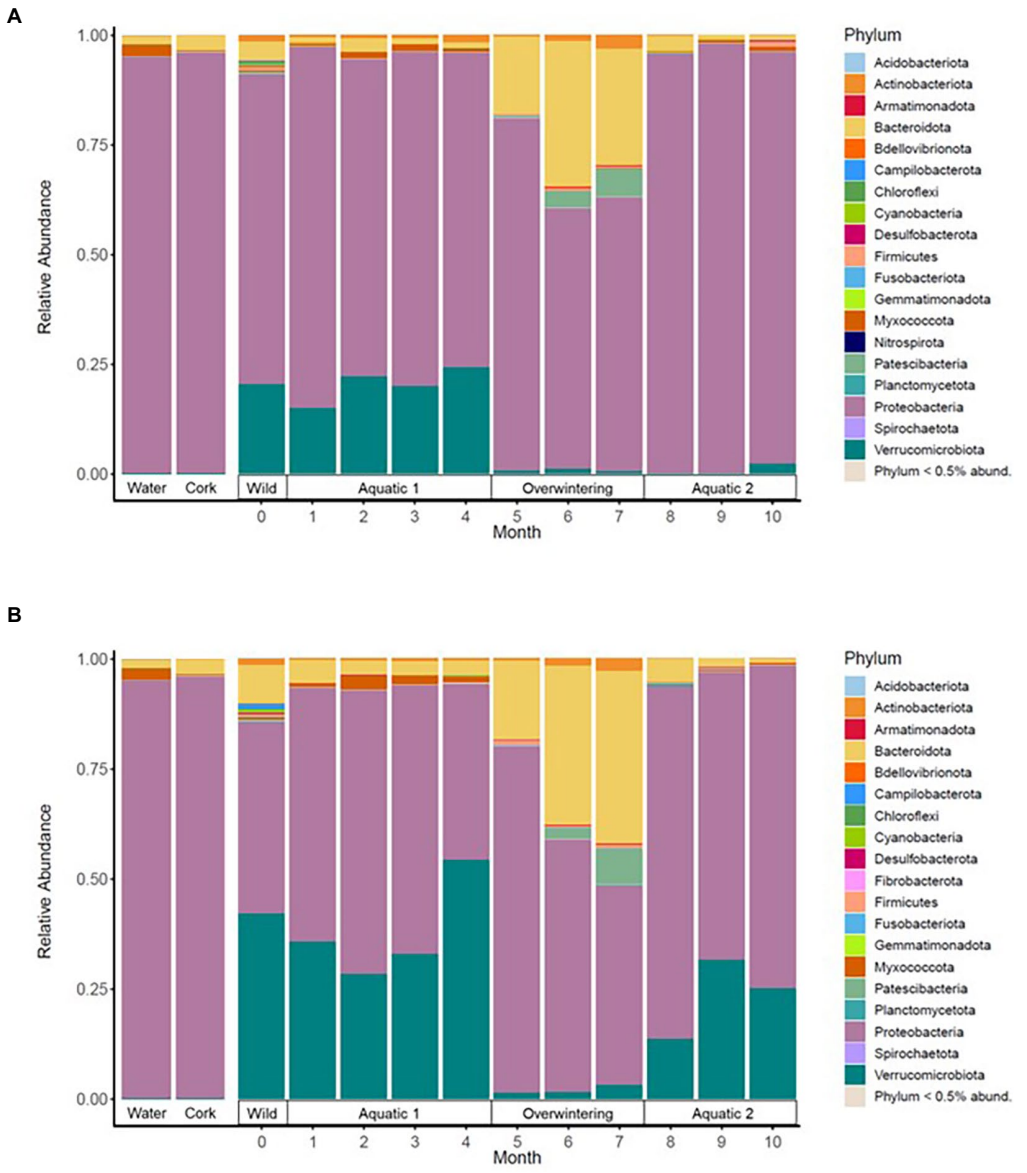
Mean relative abundance of the most frequent bacterial phylotypes in the skin microbiota of alpine **(A)** and palmate **(B)** newts, at the phylum level, across time of the experiment. Dominant phyla are identified in the legend. Environmental controls were sampled from the water filled in the aquaria (Water) and the pieces of cork (Cork) placed in the overwintering boxes.

**Figure 3:**
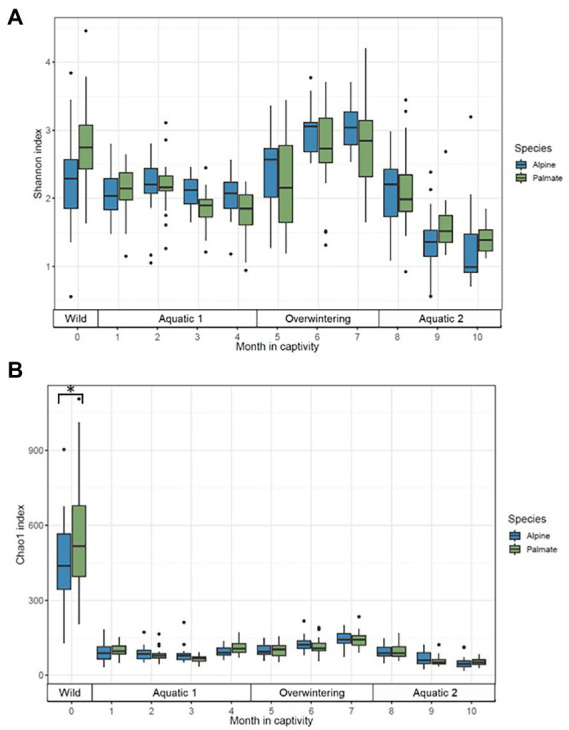
Alpha diversity of the skin microbiota of alpine (blue) and palmate (green) newts, across time of the experiment, measured as Shannon **(A)** and Chao1 **(B)** indices. Box plots represent median (horizontal line), 25th and 75th percentile (box), 5th and 95th percentile (whiskers). Dots show individual outlier values of alpha diversity. The bracket with the asterisk indicates a significant difference in diversity between wild species.

**Figure 4:**
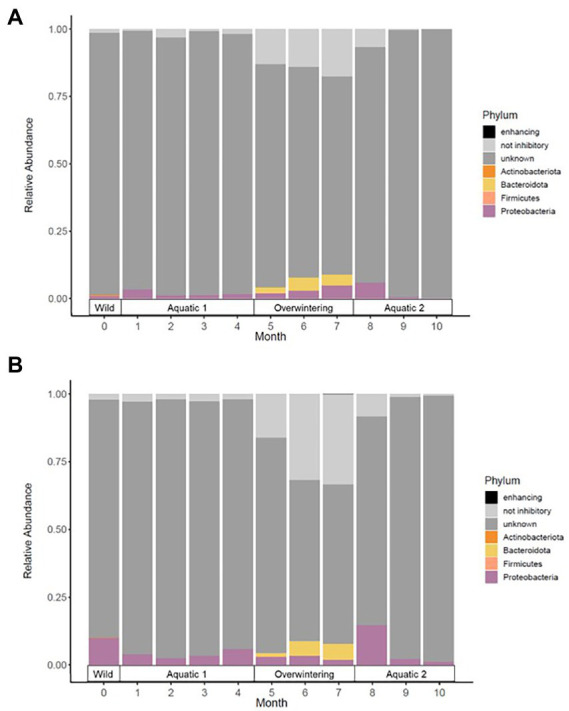
Mean relative abundance of phylotypes with known *Bd*-inhibitory activity (in color) in the skin microbiota of alpine **(A)** and palmate **(B)** newts, at the phylum level, across time of the experiment. Dominant phyla are identified in the legend. Phylotypes with no or with untested activity against the chytrid are in grey.

In both alpine and palmate newts, the skin microbiota of wild individuals was dominated by Proteobacteria, Verrucomicrobiota and Bacteroidota (Figure 2). The microbiota of palmate newts was more diverse (Shannon, *F*_(1,34)_ = 5.42, *p* = 0.026) (Figure 3A), comprised more ASVs (Supplementary Figure S1) and tended to be richer than that of alpine newts (Chao1, *F*_(1,34)_ = 2.84, *p* = 0.101) (Figure 3B). It also comprised more *Bd*-inhibitory phylotypes (Figure 4; Supplementary Figure S1). In both species, *Bd*-inhibitory phylotypes belonged to Proteobacteria, Actinobacteriota, Bacteroidota or Firmicutes phyla (Figure 4). The alpha diversity of the microbiota of wild newts was not significantly determined by their sex (Chao1, *F*_(1,34)_ = 0.34, *p* = 0.563; Shannon, *F*_(1,34)_ = 0.12 *p* = 0.735), nor by their SVL (Chao1, *F*_(1,34)_ = 0.16 *p* = 0.696; Shannon, *F*_(1,34)_ = 0.87, *p* = 0.356).

In the wild, the beta diversity of skin bacterial communities differed between species (*F*_(1,20)_ = 19.06, *p* < 0.001) and sexes (*F*_(1,20)_ = 12.03, *p* < 0.001), which explained 29 and 18% of the variation in community structure, respectively (Supplementary Figure S2), but was not significantly affected by the SVL of the newts (*F*_(1,20)_ = 0.95, *p* = 0.570). The compositional variance of microbiota did not significantly differ between species (*F*_(1,36)_ = 0.75, *p* = 0.397) nor between sexes (*F*_(1,36)_ = 0.16, *p* = 0.697). However, three ASVs, of which two Comamonadaceae and one Bacteroidales, had significantly greater relative abundance in palmate newts compared to alpine newts. One of these Comamonadaceae phylotypes has known *Bd*-inhibitory activity according to the Antifungal Isolates Database (Supplementary Table 1).

### 3.2. Effect of short-and long-term captivity on the skin microbiota

One month after their transfer into captivity, the alpha diversity of the newts’ skin microbiota had significantly decreased (Chao1, *F*_(1,39)_ = 288.04, *p* < 0.001; Shannon, *F*_(1,39)_ = 12.41, *p* = 0.001). Differences between newt species in evenness of their bacterial communities persisted (Shannon, *F*_(1,39)_ = 5.20, *p* = 0.028) while their richness remained comparable (Chao1, *F*_(1,38)_ = 3.66, *p* = 0.063) (Figure 3). The transfer into captivity also led to a 10-fold decrease in the total number of ASVs in both newt populations despite the acquisition of new taxa; after 1 month *ex-situ*, these new taxa outnumbered the ASVs retained from the wild microbiota (Supplementary Figure S3). The majority of these new bacteria in the microbiota of captive newts were absent from the water filling their aquaria, and most of the exogenous phylotypes introduced through this medium did not colonize their skin microbiota (Supplementary Figure S4A). More *Bd*-inhibitory phylotypes were lost than acquired during the transfer (ALP, 58 lost vs. 12 new; PAL, 74 lost vs. 8 new), with only 9 and 11 protective phylotypes retained in the populations of alpine and palmate newts after 1 month *ex-situ*, respectively (Supplementary Figure S3). Consequently, the species differences in relative abundance of *Bd*-inhibitory phylotypes observed in the wild (ALP, 1.4%; PAL, 10%) decreased in captivity (ALP, 3.4%; PAL, 4.0%) (Figure 4).

**Figure 5:**
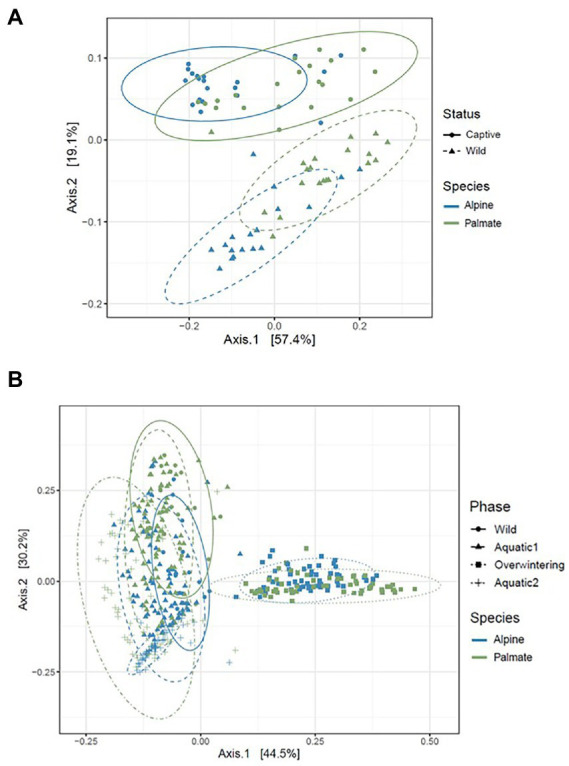
PCoA representing the beta diversity (calculated as the weighted Unifrac distance) of the skin microbiota of alpine and palmate newts, sampled in the wild and after 1  month in captivity **(A)**, and throughout all experimental phases **(B)**. Each dot represents the diversity of a microbiota sample. The species of each newt is indicated by the hue of its datapoint, and the phase of sampling is indicated by the shape of the datapoint as well as the line-type of the ellipse.

**Figure 6:**
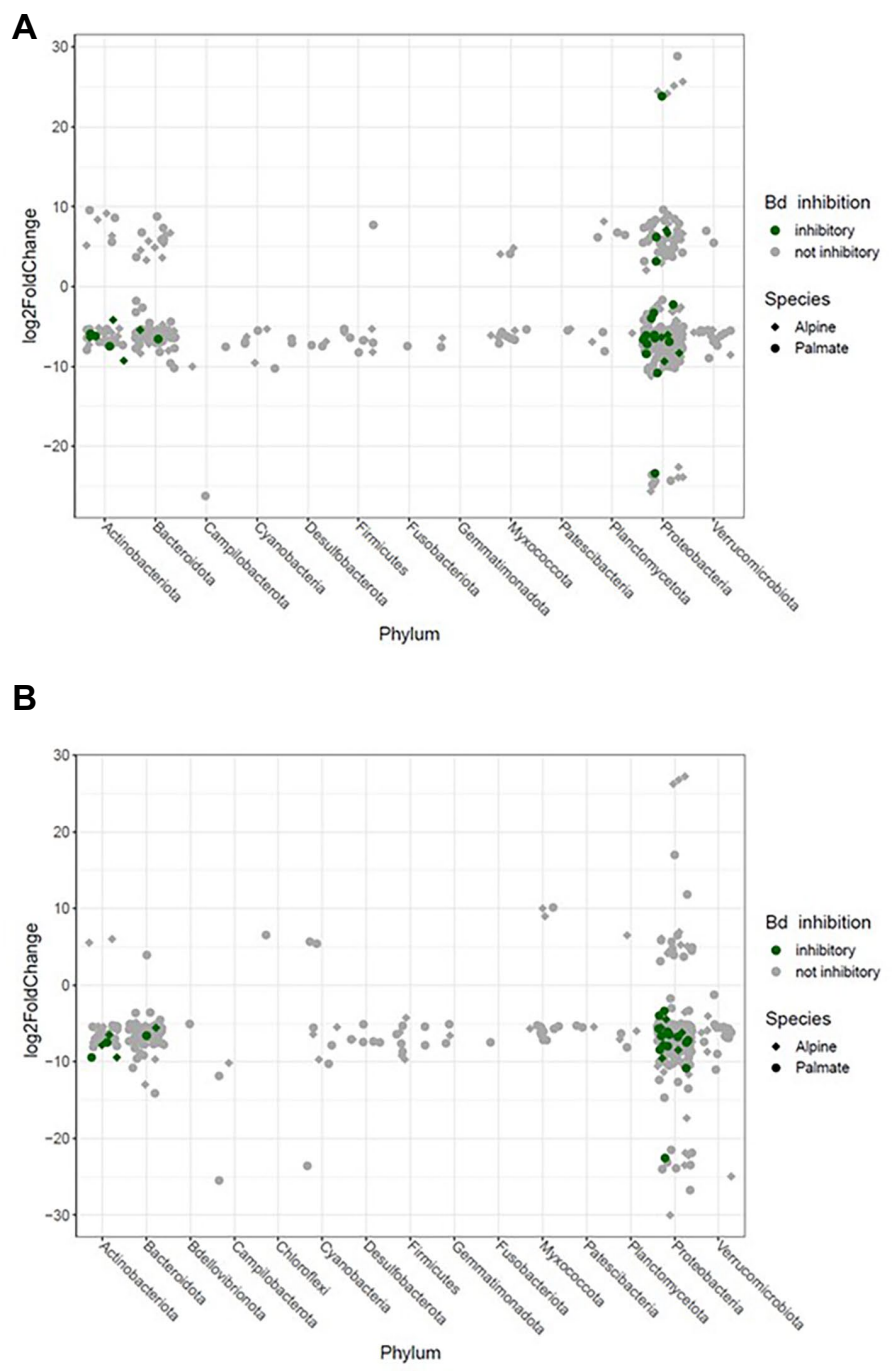
Significant log2 fold changes in abundance of bacterial phylotypes in the skin microbiota of newts over their transfer from the wild into captivity on the short term (1  month) **(A)** and longer term (10  months) **(B)**. Each phylotype is represented by a point whose position indicates the change in relative abundance throughout the transition, and whose shape characterizes the newt species in which it significantly varies in abundance. The color code indicates potential protective function of these phylotypes against *Bd*.

The beta diversity of the newts’ skin bacterial communities significantly changed after their transfer into captivity (*F*_(1,36)_ = 22.66, *p* < 0.001), and was differentially affected by that transfer among newt species (*F*_(1,36)_ = 3.19, *p* = 0.034). While 19% of the variation in community structure among samples from the wild and the first month in captivity was explained by this environmental change, 18% was still due to significant differences between species (*F*_(1,36)_ = 21.53, *p* < 0.001) (Figure 5A). The structure of the microbiota was not significantly different between individuals (*F*_(38,36)_ = 0.87, *p* = 0.770) and its compositional variance was homogenous across species (*F*_(1,76)_ = 0.08, *p* = 0.776) and between wild and captive newts (*F*_(1,76)_ = 0.81, *p* = 0.373). The transfer into captivity led to rapid changes in the relative abundance of ASVs belonging to 12 and 13 different bacterial phyla, in alpine and palmate newts, respectively. In both species, the relative abundance of Proteobacteria increased while the proportion of Bacteroidota and Verrucomicrobiota decreased (Figure 2). More specifically in alpine newts, 151 ASVs (of which eight *Bd*-inhibitory taxa) had a significantly greater relative abundance in wild individuals, while 44 (of which two *Bd*-inhibitory taxa) were more abundant after 1 month in captivity. In palmate newts, 218 phylotypes (of which 16 *Bd*-inhibitory taxa) had greater relative abundance in the wild, and 49 (of which three *Bd*-inhibitory taxa) were more abundant in captivity (Figure 6A). Overall, 117 of the decreasing ASVs and 20 of the increasing phylotypes were common to both species, and were similarly affected by this transition from the wild to captivity in this relatively short timeframe (Supplementary Tables S2, S3).

The comparison of samples from the wild and after 10 months of captivity revealed an even stronger effect of long-term captivity, with significant changes in the relative abundance of 189 and 269 ASVS, across 12 and 14 phyla in the microbiota of alpine and palmate newts, respectively. Overall, 175 phylotypes decreased in abundance in the microbiota of alpine newts, while 14 increased. In palmate newts, 253 phylotypes were significantly less abundant after 10 months in captivity, while 16 had increased in abundance. All of these increased phylotypes remain untested against *Bd*. Among ASVs that significantly decreased in abundance, 16 and 10 had known *Bd*-inhibitory activity in alpine and palmate newts, respectively (Figure 6B; Supplementary Tables S11, S12).

### 3.3. Influence of artificial aquatic and terrestrial phase-shifts on the microbiota of captive newts

Phase-shifts caused significant changes in the alpha diversity of microbial communities (Chao1, *F*_(3,7)_ = 19.55, *p* < 0.001; Shannon, *F*_(3,7)_ = 8.03, *p* = 0.011); it was generally high in the wild, decreased through captive aquatic phases, and increased during the overwintering (Figure 3). Overall, the diversity of the microbiota was not significantly different between host species (Chao1, *F*_(1,18)_ = 0.52, *p* = 0.482; Shannon, *F*_(1,23)_ = 1.04, *p* = 0.318). Microbiota were richer in wild individuals compared to any captive period (Supplementary Table S4), but their evenness was not uniformly affected by phase-shifts between the two species (Shannon, *F*_(3,403)_ = 9.05, *p* < 0.001) (Supplementary Table S5).

**Figure 7:**
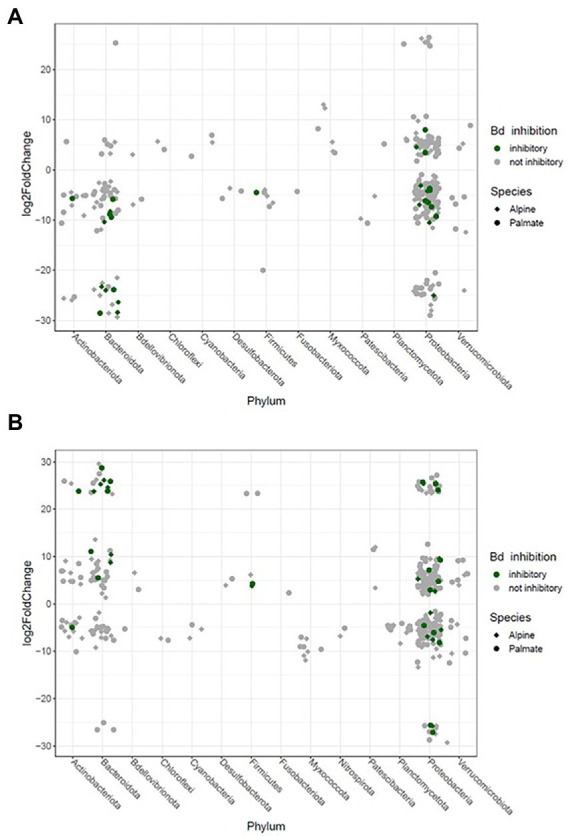
Significant log2 fold changes in abundance of bacterial phylotypes in the skin microbiota of captive newts over phase shifts from the first aquatic phase to the overwintering period **(A)** and from the overwintering period to the second aquatic phase **(B)**. Each phylotype is represented by a point whose position indicates the change in relative abundance throughout the transition, and whose shape characterizes the newt species in which it significantly varies in abundance. The color code indicates potential protective function of these phylotypes against *Bd*.

The beta diversity of bacterial communities significantly differed between aquatic and terrestrial phases, with phase-shifts explaining over 51% of the variation in community structure among samples (*F*_(3,390)_ = 179.85, *p* < 0.001) (Figure 5B). Across the 10 months of captivity, the beta diversity of the newts’ microbiota also significantly differed between species (explaining 3.9% of the variation; *F*_(1,390)_ = 41.43, *p* < 0.001), between individuals (*F*_(38,390)_ = 1.32, *p* = 0.014), and was differentially affected by phase-shifts among species (*F*_(3,390)_ = 9.70, *p* < 0.001). In both alpine and palmate newts, each phase was associated with distinct community structures (Supplementary Table S6) although Proteobacteria remained dominant throughout the 10 months of monitoring (Figure 2). Moreover, the interindividual variance in microbiota structure was not homogenous among phases and was significantly higher during the overwintering phase (*F*_(3,432)_ = 10.90, *p* < 0.001). In both species, the initially large proportion of Verrucomicrobiota in samples from the first aquatic phase decreased suddenly over the transition to the overwintering period. Conversely, Bacteroidota rapidly increased in proportion, as well as Actinobacteriota and Patescibacteria after a longer latency. These two latter phyla were present in negligible abundance on the cork from the overwintering containers (Figure 2), and only seven of the new phylotypes that colonized the newts’ microbiota during the overwintering may have come from that material (Supplementary Figure S4B). More precisely, differential abundance analyses revealed that the transition from the first aquatic phase to the overwintering period was coupled with significant changes in the abundance of 193 ASVs across 13 bacterial phyla in alpine newts, and of 191 ASVs across 14 different bacterial phyla in palmate newts. In both species, the number of phylotypes that significantly increased and decreased in abundance over this phase shift was similar. However, twice as many *Bd*-inhibitory phylotypes increased than decreased in abundance over this shift (Figure 7A; Supplementary Tables 7–8), and overwintering was therefore associated with higher abundance of *Bd*-inhibitory ASVs in both species (Figure 4). During the transition from the overwintering to the second aquatic phase, 107 and 108 phylotypes significantly decreased in abundance, while 37 and 48 ASVs increased in abundance in alpine and palmate newts, respectively. *Bd*-inhibitory phylotypes were affected similarly, and therefore decreased in abundance over that phase-shift, although this transition was more marked in alpine than palmate newts (Figure 4). The same phyla as in the shift from the first aquatic phase to the overwintering phase were involved, with the exception of an ASV from the Nitrospirota phylum (Figure 7B; Supplementary Tables S9, S10).

## 4. Discussion

This study contributes to our understanding of the effect of conservation interventions on animal microbiota. To our knowledge, this is the first investigation of the long-term effect of a transfer from the wild to captivity, using standard husbandry protocols from survival assurance populations, on amphibian skin bacterial communities. Our results show that the richness and diversity of the skin microbiota rapidly decrease when amphibians are placed in captivity, and that artificially-induced seasonal habitat-shifts implemented as part of husbandry protocols elicit important turnover among their skin bacteria. Altogether, our findings suggest that *ex-situ* conservation might impair amphibian skin microbiota.

### 4.1. Species differences in microbial composition and susceptibility to disease

The dominant phyla identified within our samples are typically present in amphibian skin microbiota (Kueneman et al., 2014; Bletz et al., 2016; García-Sánchez et al., 2022). Despite being phylogenetically close (Zhang et al., 2008; Recuero et al., 2014) and inhabiting the same pond in the wild, alpine and palmate newts had distinct skin bacterial communities. Species-specific variation in microbiota composition is often reported in cohabiting amphibians (Kueneman et al., 2014; Walke et al., 2014) and the natural differences in community structure between species could explain their different susceptibility to skin diseases. Indeed, previous research suggests that palmate newts may be tolerant to *Bd*, while this pathogen can be lethal to alpine newts (Cheatsazan et al., 2013; Miaud et al., 2016). Our results show that in the wild, palmate newts had a larger number of different bacterial phylotypes, a higher alpha diversity, and a higher proportion of *Bd*-inhibitory bacteria than alpine newts. Moreover, two Comamonadaceae phylotypes were significantly more abundant in palmate newts; several isolates in this family inhibit *Bd in-vitro* (Walke et al., 2015) and one of the ASVs identified here has known *Bd*-inhibitory activity (Woodhams et al., 2015). Differences in abundance of these phylotypes should be tested in other populations to confirm whether they explain the distinct susceptibilities of alpine and palmate newts to *Bd*.

### 4.2. Rapid reorganization of the newts’ microbiota following transfer into captivity

Despite initial differences in composition and diversity of their bacterial communities in the wild, both host species were similarly affected by their transfer into captivity. After only 1 month *ex-situ*, the newts had a different microbiota structure than in the wild. Studies comparing wild and captive-reared amphibians (Becker et al., 2014; Sabino-Pinto et al., 2016; Kueneman et al., 2016b) or other vertebrates (Dallas and Warne, 2022) similarly report distinct microbial communities between individuals living *in-and ex-situ*. The diversity of the microbiota of both alpine and palmate newts strongly decreased upon arrival in captivity, corroborating findings from shorter-term captivity studies (Bates et al., 2019) and comparisons of wild amphibians to conspecific individuals maintained *ex-situ* for several generations (Sabino-Pinto et al., 2016; Passos et al., 2018). Interestingly, the proportion of *Bd*-inhibitory phylotypes was not specifically affected by captivity and while a few new protective bacteria were acquired *ex-situ*, they did not become significantly abundant over the 10 months of captivity. Similar results were reported in red-backed salamanders, which conserve constant proportions of the symbiont *Janthinobacterium lividum* when transferred into captivity (Loudon et al., 2014). Conversely, the proportion of Proteobacteria in the microbiota of the newts strongly increased after only 1 month in captivity. Elevated abundance of this ubiquitous bacterial phylum (Kersters et al., 2006) is reported in other studies comparing captive amphibians to wild conspecifics (Bataille et al., 2016; Bates et al., 2019).

Overall, little of the wild microbiota was retained through the transfer into captivity, and the microbial community of captive newts comprised a reduced number of different phylotypes than when they were in the wild. This corroborates findings that the microbiota of amphibians maintained in a biosecure collection for several generations was less rich than that of wild individuals (Passos et al., 2018). Interestingly, most phylotypes present in the microbiota of the newts after 1 month in captivity were new and of unknown origin, despite our biosecurity protocol. Although the water filling their aquaria may have introduced up to 83 exogenous bacteria, most phylotypes present in the water did not colonize their microbiota. Indeed, despite being largely assembled from environmental reservoirs of bacteria in the wild (Walke et al., 2014), the amphibian skin microbiota may not select for most microbes present *ex-situ*. This is supported by findings that the microbiota of African clawed frogs reared under standard and sterile husbandry protocols have similar diversity indices (Piccinni et al., 2021). However, it should be noted that swab-sampling of the water and cork as we did is too restrictive to fully identify environmental microbiota, so the comparison with the microbiota of the newts is indicative rather than comprehensive.

### 4.3. Restructuration of skin microbiota over artificially-induced habitat-shifts

Seasonal shifts in the structure of skin microbiota are reported in wild amphibians (Longo et al., 2015; Tong et al., 2020; Douglas et al., 2021) but to our knowledge, they have never been explored under artificial settings *ex-situ*. We found that although alpine and palmate newts maintained distinguishable skin bacterial communities throughout the 10 months of monitoring, they were similarly affected by the transfer into captivity and the artificial phase-shifts. Their alpha diversity, which had strongly diminished upon arrival in captivity, significantly increased during overwintering. In the wild, this phase has been associated with various effects on the alpha diversity of skin microbiota depending on host species (Longo et al., 2015; Tong et al., 2020; Douglas et al., 2021). Despite reestablishing similar levels of alpha diversity as in the wild, overwintering was associated with a very different microbial community structure and a much higher compositional variance than other phases. The segregation of microbiota structures was stronger between phases than it was between newt species, suggesting a similar and strong effect of artificial phase-shifts on the beta diversity of both species. Indeed, terrestrial and freshwater ecosystems, occupied during overwintering and active phases respectively, are generally associated with distinct microbial environments (Thompson et al., 2017). Moreover, seasonal variation in skin structure and feeding activity in amphibians can indirectly affect their microbiota, as diet (Antwis et al., 2014), sloughing (Meyer et al., 2012) and skin morphology (Kueneman et al., 2014) are known to influence their skin bacterial communities. Lastly, interactions among microbes also likely participated in shaping microbiota throughout phase-shifts, although this could not be further explored in this study.

Interestingly, while a few studies comparing different species *in-and ex-situ* have reported an among-individual uniformization of the microbiota in captivity (Becker et al., 2014; Hernández-Gómez et al., 2019; Edenborough et al., 2020), an individual signature in the structure of the microbiota persisted throughout phase-shifts in our experimental newts. Despite the significant decrease in alpha diversity and in abundance of many phylotypes over the 10 months of monitoring, the heterogeneity of bacterial communities between individuals was not reduced by captivity. Moreover, the variation in composition of the microbiota of captive newts was hardly influenced by exogenous bacteria in their *ex-situ* environment. For example, only seven of the phylotypes acquired in the overwintering phase were common to the cork substrate filling their containers. Moreover, changes in the relative abundance of major phyla in the microbiota of the newts throughout phase-shifts revealed a strong selection for bacterial taxa associated with each phase. We observed an increase in proportion of Bacteroidota, Actinobacteriota and of antifungal ASVs during the overwintering terrestrial phase, which are also reported in wild individuals during this period of elevated prevalence of *Bd* (Tong et al., 2020; Douglas et al., 2021; Le Sage et al., 2021).

### 4.4. Preserving microbial communities to improve the *ex-situ* conservation of their hosts

Several authors encouraged the integration of microbiota to conservation plans developed for their hosts (Trevelline et al., 2019; West et al., 2019; Carthey et al., 2020), and this approach would be particularly relevant for amphibians given the essential role of their bacterial symbionts against emerging diseases (Vredenburg et al., 2011; Rebollar et al., 2020). Indeed, microbiota richness is associated with *Bd* inhibition (Piovia-Scott et al., 2017), and generally, highly diverse microbiota seem beneficial to the health of their host (Longo et al., 2015; Bates et al., 2018; Harrison et al., 2019). Yet, our results show that current management strategies such as *ex-situ* conservation and artificial habitat-shifts alter amphibian skin bacterial communities, which could consequently be detrimental to their host and reduce the success of reintroduction efforts. Functional analyses and transcriptomics should now be implemented to characterize the implications of these shifts in microbiota structure for their amphibian hosts.

Although bioaugmentation with probiotics can successfully limit diversity loss in microbiota of captive animals (Bletz et al., 2013; Woodhams et al., 2016), this strategy does not perpetuate exact replica of natural bacterial communities, and thus cannot prevent the elimination of phylotypes associated with specific metabolic pathways, which could affect their host functional resilience or put them at higher risk of infection by pathogens if reintroduced in the wild (Dallas and Warne, 2022). “Rewilding” the microbiota of captive animals by placing them in outdoor mesocosms before reintroduction in the wild may be a more promising solution, but should be investigated further (Kueneman et al., 2022). Considering the accelerating decline rate of amphibians (McCallum, 2007), it is critical to integrate microbiota to amphibian applied conservation and to continue to develop methods to maintain wild microbiota in captivity. More generally, the effect of conservation approaches on the microbiota of endangered species should be closely investigated, as they can be considered as an overlooked form of anthropogenic disturbance.

## Data availability statement

The datasets presented in this study can be found in online repositories. The names of the repository/repositories and accession number(s) can be found at: https://figshare.com/; https://figshare.com/s/bcd8e0fe75f28c7c571b.

## Ethics statement

The animal study was reviewed and approved by our capture permit was issued by the Direction Régionale de l’Environnement, de l’Aménagement et du Logement (DREAL) d’Occitanie. All animal care, husbandry and experimental procedures were approved by the Animal Ethics Commission of the University of Liège (protocol 1613) and were conducted in an accredited laboratory (LA1610429).

## Author contributions

LFM, DL, and MD conceived ideas and methodology. MD and DL supervised the study. MB, LFM, DL, and MD acquired funding. LFM maintained the *ex-situ* collection and collected swab samples with help from MD and MB. LFM and KH conducted lab-work. LFM and PL conducted data analysis. LFM wrote the first draft of the manuscript. All authors worked on the subsequent drafts and approved the final version of the manuscript.

## Funding

This research was funded by the European Union of Aquarium Curators and by an NSERC Discovery Grant (RGPIN/6877-2018) to DL. LFM is a PhD student funded by the ReNewZoo program through an NSERC Create Grant. MD is a Research Director at F.R.S.-FNRS (Fonds de la Recherche Scientifique).

## Conflict of interest

The authors declare that the research was conducted in the absence of any commercial or financial relationships that could be construed as a potential conflict of interest.

## Publisher’s note

All claims expressed in this article are solely those of the authors and do not necessarily represent those of their affiliated organizations, or those of the publisher, the editors and the reviewers. Any product that may be evaluated in this article, or claim that may be made by its manufacturer, is not guaranteed or endorsed by the publisher.

## Footnotes

^1^ https://figshare.com/s/bcd8e0fe75f28c7c571b

## References

[ref1] Antwis R. E. Haworth R. L. Engelmoer D. J. P. Ogilvy V. Fidgett A. L. Preziosi R. F. (2014). Ex situ diet influences the bacterial community associated with the skin of red-eyed tree frogs (*Agalychnis callidryas*). PLoS One 9:e85563. doi: 10.1371/journal.pone.0085563, PMID: 24416427 PMC3887054

[ref2] Bataille A. Lee-Cruz L. Tripathi B. Kim H. Waldman B. (2016). Microbiome variation across amphibian skin regions: implications for chytridiomycosis mitigation efforts. Microb. Ecol. 71, 221–232. doi: 10.1007/s00248-015-0653-0, PMID: 26271741

[ref3] Bates K. A. Clare F. C. O’Hanlon S. Bosch J. Brookes L. Hopkins K.. (2018). Amphibian chytridiomycosis outbreak dynamics are linked with host skin bacterial community structure. Nat. Commun. 9:693. doi: 10.1038/s41467-018-02967-w, PMID: 29449565 PMC5814395

[ref4] Bates K. A. Shelton J. M. G. Mercier V. L. Hopkins K. P. Harrison X. A. Petrovan S. O.. (2019). Captivity and infection by the fungal pathogen *Batrachochytrium salamandrivorans* perturb the amphibian skin microbiome. Front. Microbiol. 10:1834. doi: 10.3389/fmicb.2019.01834, PMID: 31507541 PMC6716147

[ref5] Beck C. W. Bliwise N. G. (2014). Interactions are critical. CBE—Life Sci. Educ. 13, 371–372. doi: 10.1187/cbe.14-05-0086, PMID: 25185220 PMC4152198

[ref6] Becker M. H. Richards-Zawacki C. L. Gratwicke B. Belden L. K. (2014). The effect of captivity on the cutaneous bacterial community of the critically endangered Panamanian golden frog (*Atelopus zeteki*). Biol. Conserv. 176, 199–206. doi: 10.1016/j.biocon.2014.05.029

[ref7] Bird A. K. Prado-Irwin S. R. Vredenburg V. T. Zink A. G. (2018). Skin microbiomes of California terrestrial salamanders are influenced by habitat more than host phylogeny. Front. Microbiol. 9:442. doi: 10.3389/fmicb.2018.00442, PMID: 29593686 PMC5861191

[ref8] Blaustein A. R. Han B. A. Relyea R. A. Johnson P. T. J. Buck J. C. Gervasi S. S.. (2011). The complexity of amphibian population declines: understanding the role of cofactors in driving amphibian losses. Ann. N. Y. Acad. Sci. 1223, 108–119. doi: 10.1111/j.1749-6632.2010.05909.x, PMID: 21449968

[ref9] Bletz M. C. Goedbloed D. J. Sanchez E. Reinhardt T. Tebbe C. C. Bhuju S.. (2016). Amphibian gut microbiota shifts differentially in community structure but converges on habitat-specific predicted functions. Nat. Commun. 7:13699. doi: 10.1038/ncomms13699, PMID: 27976718 PMC5171763

[ref10] Bletz M. C. Loudon A. H. Becker M. H. Bell S. C. Woodhams D. C. Minbiole K. P.. (2013). Mitigating amphibian chytridiomycosis with bioaugmentation: characteristics of effective probiotics and strategies for their selection and use. Ecol. Lett. 16, 807–820. doi: 10.1111/ele.12099, PMID: 23452227

[ref11] Bletz M. C. Perl R. G. Bobowski B. T. Japke L. M. Tebbe C. C. Dohrmann A. B.. (2017). Amphibian skin microbiota exhibits temporal variation in community structure but stability of predicted Bd-inhibitory function. ISME J. 11, 1521–1534. doi: 10.1038/ismej.2017.41, PMID: 28387770 PMC5520157

[ref12] Bokulich N. A. Subramanian S. Faith J. J. Gevers D. Gordon J. I. Knight R.. (2013). Quality-filtering vastly improves diversity estimates from Illumina amplicon sequencing. Nat. Methods 10, 57–59. doi: 10.1038/nmeth.2276, PMID: 23202435 PMC3531572

[ref13] Broza M. Halpern M. (2001). Chironomid egg masses and *Vibrio cholerae*. Nature 412:40. doi: 10.1038/35083691, PMID: 11452294

[ref14] Brucker R. M. Harris R. N. Schwantes C. R. Gallaher T. N. Flaherty D. C. Lam B. A.. (2008). Amphibian chemical defense: antifungal metabolites of the microsymbiont *Janthinobacterium lividum* on the salamander *Plethodon cinereus*. J. Chem. Ecol. 34, 1422–1429. doi: 10.1007/s10886-008-9555-7, PMID: 18949519

[ref15] Calatayud N. E. Hammond T. T. Gardner N. R. Curtis M. J. Swaisgood R. R. Shier D. M. (2021). Benefits of overwintering in the conservation breeding and translocation of a critically endangered amphibian. Conserv. Sci. Pract. 3:e341. doi: 10.1111/csp2.341

[ref16] Callahan B. J. McMurdie P. J. Rosen M. J. Han A. W. Johnson A. J. A. Holmes S. P. (2016). DADA2: high-resolution sample inference from Illumina amplicon data. Nat. Methods 13, 581–583. doi: 10.1038/nmeth.3869, PMID: 27214047 PMC4927377

[ref17] Cameron E. S. Schmidt P. J. Tremblay B. J.-M. Emelko M. B. Müller K. M. (2021). Enhancing diversity analysis by repeatedly rarefying next generation sequencing data describing microbial communities. Sci. Rep. 11:22302. doi: 10.1038/s41598-021-01636-1, PMID: 34785722 PMC8595385

[ref18] Carthey A. J. R. Blumstein D. T. Gallagher R. V. Tetu S. G. Gillings M. R. (2020). Conserving the holobiont. Funct. Ecol. 34, 764–776. doi: 10.1111/1365-2435.13504

[ref19] Cheatsazan H. de Almedia A. P. L. G. Russell A. F. Bonneaud C. (2013). Experimental evidence for a cost of resistance to the fungal pathogen, *Batrachochytrium dendrobatidis*, for the palmate newt, *Lissotriton helveticus*. BMC Ecol. 13:27. doi: 10.1186/1472-6785-13-27, PMID: 23866033 PMC3722082

[ref20] Dallas J. W. Warne R. W. (2022). Captivity and animal microbiomes: potential roles of microbiota for influencing animal conservation. Microb. Ecol. 1–19. doi: 10.1007/s00248-022-01991-0, PMID: 35316343

[ref21] Davis N. M. Proctor D. M. Holmes S. P. Relman D. A. Callahan B. J. (2018). Simple statistical identification and removal of contaminant sequences in marker-gene and metagenomics data. Microbiome 6:226. doi: 10.1186/s40168-018-0605-2, PMID: 30558668 PMC6298009

[ref22] Dawson J. Patel F. Griffiths R. A. Young R. P. (2016). Assessing the global zoo response to the amphibian crisis through 20-year trends in captive collections. Conserv. Biol. 30, 82–91. doi: 10.1111/cobi.12563, PMID: 26219401

[ref23] Douglas A. E. (2018). Fundamentals of Microbiome Science: How Microbes Shape Animal Biology. Princeton, New Jersey, USA: Princeton University Press.

[ref24] Douglas A. J. Hug L. A. Katzenback B. A. (2021). Composition of the north American wood frog (*Rana sylvatica*) bacterial skin microbiome and seasonal variation in community structure. Microb. Ecol. 81, 78–92. doi: 10.1007/s00248-020-01550-5, PMID: 32613267

[ref25] Edenborough K. M. Mu A. Mühldorfer K. Lechner J. Lander A. Bokelmann M.. (2020). Microbiomes in the insectivorous bat species Mops condylurus rapidly converge in captivity. PLoS One 15:e0223629. doi: 10.1371/journal.pone.0223629, PMID: 32196505 PMC7083271

[ref26] Gao C.-H. Yu G. Cai P. (2021). ggVennDiagram: an intuitive, easy-to-use, and highly customizable R package to generate Venn diagram. Front. Genet. 12:706907. doi: 10.3389/fgene.2021.706907, PMID: 34557218 PMC8452859

[ref27] García-Sánchez J. C. Arredondo-Centeno J. Segovia-Ramirez M. G. Tenorio Olvera A. M. Parra-Olea G. Vredenburg V. T.. (2022). Factors influencing bacterial and fungal skin communities of montane salamanders of Central Mexico. Microb. Ecol. 1–17. doi: 10.1007/s00248-022-02049-x, PMID: 35705744

[ref28] Gascon C. (2007). Amphibian Conservation Action Plan: Proceedings of the IUCN/SSC Amphibian Conservation Summit 2005. Gland, Switzerland: IUCN-the World Conservation Union.

[ref29] Gołębiewski M. Tretyn A. (2020). Generating amplicon reads for microbial community assessment with next-generation sequencing. J. Appl. Microbiol. 128, 330–354. doi: 10.1111/jam.14380, PMID: 31299126

[ref30] Gray M. J. Spatz J. A. Carter E. D. Yarber C. M. Wilkes R. P. Miller D. L. (2018). Poor biosecurity could lead to disease outbreaks in animal populations. PLoS One 13:e0193243. doi: 10.1371/journal.pone.0193243, PMID: 29513691 PMC5841743

[ref31] Griffiths R. A. Pavajeau L. (2008). Captive breeding, reintroduction, and the conservation of amphibians. Conserv. Biol. 22, 852–861. doi: 10.1111/j.1523-1739.2008.00967.x, PMID: 18616746

[ref32] Harding G. Griffiths R. A. Pavajeau L. (2016). Developments in amphibian captive breeding and reintroduction programs. Conserv. Biol. 30, 340–349. doi: 10.1111/cobi.12612, PMID: 26306460

[ref33] Harrison X. A. Price S. J. Hopkins K. Leung W. T. M. Sergeant C. Garner T. W. J. (2019). Diversity-stability dynamics of the amphibian skin microbiome and susceptibility to a lethal viral pathogen. Front. Microbiol. 10:2883. doi: 10.3389/fmicb.2019.02883, PMID: 31956320 PMC6951417

[ref34] Hernández-Gómez O. Briggler J. T. Williams R. N. (2019). Captivity-induced changes in the skin microbial communities of hellbenders (*Cryptobranchus alleganiensis*). Microb. Ecol. 77, 782–793. doi: 10.1007/s00248-018-1258-1, PMID: 30209587

[ref35] IUCN (2022). The IUCN red list of threatened species. Available at: https://www.iucnredlist.org/es

[ref36] Jensen M. Jensen U. Bertelsen M. (2021). Assessing the effects of biosecurity measures in terrarium management. J. Zoo Aquar. Res. 9, 157–160. doi: 10.19227/jzar.v9i3.470

[ref37] Kaehler B. D. Bokulich N. A. McDonald D. Knight R. Caporaso J. G. Huttley G. A. (2019). Species abundance information improves sequence taxonomy classification accuracy. Nat. Commun. 10:4643. doi: 10.1038/s41467-019-12669-6, PMID: 31604942 PMC6789115

[ref38] Kassambara A. (2019). Ggpubr: 'ggplot2' based publication ready plots. R package version 0.2. Available at: https://CRAN.R-project.org/package=ggpubr

[ref39] Kersters K. De Vos P. Gillis M. Swings J. Vandamme P. Stackebrandt E. R. (2006). “Introduction to the Proteobacteria” in The Prokaryotes: A Handbook on the Biology of Bacteria. eds. Dworkin M. Falkow S. Rosenberg E. Schleifer K. H. Stackebrandt E. (NY: Springer), 3–37.

[ref40] Kueneman J. G. Bletz M. C. Becker M. Gratwicke B. Garcés O. A. Hertz A.. (2022). Effects of captivity and rewilding on amphibian skin microbiomes. Biol. Conserv. 271:109576. doi: 10.1016/j.biocon.2022.109576

[ref41] Kueneman J. G. Parfrey L. W. Woodhams D. C. Archer H. M. Knight R. McKenzie V. J. (2014). The amphibian skin-associated microbiome across species, space and life history stages. Mol. Ecol. 23, 1238–1250. doi: 10.1111/mec.12510, PMID: 24171949

[ref42] Kueneman J. G. Woodhams D. C. Van Treuren W. Archer H. M. Knight R. McKenzie V. J. (2016a). Inhibitory bacteria reduce fungi on early life stages of endangered Colorado boreal toads (*Anaxyrus boreas*). ISME J. 10, 934–944. doi: 10.1038/ismej.2015.168, PMID: 26565725 PMC4796932

[ref43] Kueneman J. G. Woodhams D. C. Harris R. Archer H. M. Knight R. McKenzie V. J. (2016b). Probiotic treatment restores protection against lethal fungal infection lost during amphibian captivity. Proc. R. Soc. B Biol. Sci. 283:20161553. doi: 10.1098/rspb.2016.1553, PMID: 27655769 PMC5046908

[ref44] Le Sage E. H. LaBumbard B. C. Reinert L. K. Miller B. T. Richards-Zawacki C. L. Woodhams D. C.. (2021). Preparatory immunity: seasonality of mucosal skin defences and *Batrachochytrium* infections in southern leopard frogs. J. Anim. Ecol. 90, 542–554. doi: 10.1111/1365-2656.13386, PMID: 33179786

[ref45] Longo A. V. Savage A. E. Hewson I. Zamudio K. R. (2015). Seasonal and ontogenetic variation of skin microbial communities and relationships to natural disease dynamics in declining amphibians. R. Soc. Open Sci. 2:140377. doi: 10.1098/rsos.140377, PMID: 26587253 PMC4632566

[ref46] Loudon A. H. Woodhams D. C. Parfrey L. W. Archer H. Knight R. McKenzie V.. (2014). Microbial community dynamics and effect of environmental microbial reservoirs on red-backed salamanders (*Plethodon cinereus*). ISME J. 8, 830–840. doi: 10.1038/ismej.2013.200, PMID: 24335825 PMC3960541

[ref47] Love M. I. Huber W. Anders S. (2014). Moderated estimation of fold change and dispersion for RNA-seq data with DESeq2. Genome Biol. 15:550. doi: 10.1186/s13059-014-0550-8, PMID: 25516281 PMC4302049

[ref48] Lozupone C. Lladser M. E. Knights D. Stombaugh J. Knight R. (2011). UniFrac: an effective distance metric for microbial community comparison. ISME J. 5, 169–172. doi: 10.1038/ismej.2010.133, PMID: 20827291 PMC3105689

[ref49] McCallum M. L. (2007). Amphibian decline or extinction? Current declines dwarf background extinction rate. J. Herpetol. 41, 483–491. doi: 10.1670/0022-1511(2007)41[483:ADOECD]2.0.CO;2

[ref50] McMurdie P. J. Holmes S. (2013). Phyloseq: an R package for reproducible interactive analysis and graphics of microbiome census data. PLoS One 8:e61217. doi: 10.1371/journal.pone.0061217, PMID: 23630581 PMC3632530

[ref51] Mendelson J. R. (2018). “Frogs in glass boxes: responses of zoos to global amphibian extinctions” in The Ark and Beyond: The Evolution of Zoo and Aquarium conservation. eds. Minteer B. A. Maienschein J. Collins J. P. (Chicago, IL: University of Chicago Press)

[ref52] Mendelson J. R. Gagliardo F. Andreone K. R. Buley K. R. Coloma R. Garcia G.. (2007). “Captive programs” in Amphibian Conservation Action Plan: Proceedings of the IUCN/SSC Amphibian Conservation summit 2005. eds. Gascon C. Collins J. P. Moore R. D. Church D. R. McKay J. E. Mendelson J. R. (Gland, Switzerland: IUCN/SSC Amphibian Specialist Group), 36–37.

[ref53] Meyer E. A. Cramp R. L. Bernal M. H. Franklin C. E. (2012). Changes in cutaneous microbial abundance with sloughing: possible implications for infection and disease in amphibians. Dis. Aquat. Org. 101, 235–242. doi: 10.3354/dao02523, PMID: 23324420

[ref54] Miaud C. Dejean T. Savard K. Millery-Vigues A. Valentini A. Gaudin, N C. G.. (2016). Invasive north American bullfrogs transmit lethal fungus *Batrachochytrium dendrobatidis* infections to native amphibian host species. Biol. Invasions 18, 2299–2308. doi: 10.1007/s10530-016-1161-y

[ref55] Moore B. C. Martinez E. Gay J. M. Rice D. H. (2003). Survival of salmonella enterica in freshwater and sediments and transmission by the aquatic midge *Chironomus tentans* (Chironomidae: Diptera). Appl. Environ. Microbiol. 69, 4556–4560. doi: 10.1128/AEM.69.8.4556-4560.2003, PMID: 12902242 PMC169145

[ref56] Oksanen J. Blanchet G. Friendly M. Kindt R. Legendre P. McGlinn D.. (2020). Vegan: community ecology package. R package version 2.5-7. Available at: https://CRAN.R-project.org/package=vegan

[ref57] Passos L. F. Garcia G. Young R. J. (2018). Comparing the bacterial communities of wild and captive golden mantella frogs: implications for amphibian conservation. PLoS One 13:e0205652. doi: 10.1371/journal.pone.0205652, PMID: 30379861 PMC6209184

[ref58] Peixoto R. S. Harkins D. M. Nelson K. E. (2021). Advances in microbiome research for animal health. Annu. Rev. Anim. Biosci. 9, 289–311. doi: 10.1146/annurev-animal-091020-075907 33317323

[ref59] Pessier A. P. Mendelson J. R. (2010). A manual for control of infectious diseases in amphibian survival assurance colonies and reintroduction programs: proceedings from a workshop: 16–18 February 2009 San Diego zoo. IUCN/SSC Conservation Breeding Specialist Group. 21–48. Available at: https://repository.sandiegozoo.org/handle/20.500.12634/940

[ref60] Piccinni M. Z. Watts J. E. M. Fourny M. Guille M. Robson S. C. (2021). The skin microbiome of *Xenopus laevis* and the effects of husbandry conditions. Anim. Microbiome 3:17. doi: 10.1186/s42523-021-00080-w, PMID: 33546771 PMC7866774

[ref61] Piovia-Scott J. Rejmanek D. Woodhams D. C. Worth S. J. Kenny H. McKenzie V.. (2017). Greater species richness of bacterial skin symbionts better suppresses the amphibian fungal pathogen *Batrachochytrium dendrobatidis*. Microb. Ecol. 74, 217–226. doi: 10.1007/s00248-016-0916-4, PMID: 28064360

[ref62] R Core Team (2022). R: A Language and Environment for Statistical Computing. Vienna, R Foundation for Statistical Computing

[ref63] Rebollar E. Martínez-Ugalde E. Orta A. (2020). The amphibian skin microbiome and its protective role against chytridiomycosis. Herpetologica 76:167. doi: 10.1655/0018-0831-76.2.167

[ref64] Recuero E. Buckley D. García-París M. Arntzen J. W. Cogălniceanu D. Martínez-Solano I. (2014). Evolutionary history of *Ichthyosaura alpestris* (Caudata, Salamandridae) inferred from the combined analysis of nuclear and mitochondrial markers. Mol. Phylogenet. Evol. 81, 207–220. doi: 10.1016/j.ympev.2014.09.014, PMID: 25263421

[ref65] Rouf M. A. Rigney M. M. (1993). Bacterial florae in larvae of the lake fly *Chironomus plumosus*. Appl. Environ. Microbiol. 59, 1236–1241. doi: 10.1128/aem.59.4.1236-1241.1993, PMID: 16348917 PMC202267

[ref66] Sabino-Pinto J. Bletz M. C. Islam M. M. Shimizu N. Bhuju S. Geffers R.. (2016). Composition of the cutaneous bacterial community in Japanese amphibians: effects of captivity, host species, and body region. Microb. Ecol. 72, 460–469. doi: 10.1007/s00248-016-0797-6, PMID: 27278778

[ref67] Santana F. E. Swaisgood R. R. Lemm J. M. Fisher R. N. Clark R. W. (2015). Chilled frogs are hot: hibernation and reproduction of the endangered mountain yellow-legged frog *Rana muscosa*. Endanger. Species Res. 27, 43–51. doi: 10.3354/esr00648

[ref68] Scheele B. C. Pasmans F. Skerratt L. F. Berger L. Martel A. Beukema W.. (2019). Amphibian fungal panzootic causes catastrophic and ongoing loss of biodiversity. Science 363, 1459–1463. doi: 10.1126/science.aav0379, PMID: 30923224

[ref69] Silla A. J. Calatayud N. E. Trudeau V. L. (2021). Amphibian reproductive technologies: approaches and welfare considerations. Conserv. Physiol. 9:coab011. doi: 10.1093/conphys/coab011, PMID: 33763231 PMC7976225

[ref70] Species 360 (2021). Zoological information management system (ZIMS) for husbandry. Available at: www.zims.Species360.org

[ref71] Tapley B. Bradfield K. S. Michaels C. Bungard M. (2015). Amphibians and conservation breeding programmes: do all threatened amphibians belong on the ark? Biodivers. Conserv. 24, 2625–2646. doi: 10.1007/s10531-015-0966-9

[ref72] Thompson L. R. Sanders J. G. McDonald D. Amir A. Ladau J. Locey K. J.. (2017). A communal catalogue reveals Earth’s multiscale microbial diversity. Nature 551, 457–463. doi: 10.1038/nature24621, PMID: 29088705 PMC6192678

[ref73] Tong Q. Hu Z. Du X. Bie J. Wang H. (2020). Effects of seasonal hibernation on the similarities between the skin microbiota and gut microbiota of an amphibian (*Rana dybowskii*). Microb. Ecol. 79, 898–909. doi: 10.1007/s00248-019-01466-9, PMID: 31820074

[ref74] Trevelline B. K. Fontaine S. S. Hartup B. K. Kohl K. D. (2019). Conservation biology needs a microbial renaissance: a call for the consideration of host-associated microbiota in wildlife management practices. Proc. R. Soc. B Biol. Sci. 286:20182448. doi: 10.1098/rspb.2018.2448, PMID: 30963956 PMC6364583

[ref75] Van Rooij P. Pasmans F. Coen Y. Martel A. (2017). Efficacy of chemical disinfectants for the containment of the salamander chytrid fungus *Batrachochytrium salamandrivorans*. PLoS One 12:e0186269. doi: 10.1371/journal.pone.0186269, PMID: 29023562 PMC5638399

[ref76] Vredenburg V. T. Briggs C. J. Harris R. N. (2011). “Host-pathogen dynamics of amphibian chytridiomycosis: the role of the skin microbiome in health and disease”, in Fungal Diseases: An Emerging Threat to Human, Animal and Plant Health, ed. Olsen L. Choffnes E. R. Relman D. A. Pray L. (Washington (DC) National Academies Press), 342–354.

[ref77] Walke J. B. Becker M. H. Loftus S. C. House L. L. Cormier G. Jensen R. V.. (2014). Amphibian skin may select for rare environmental microbes. ISME J. 8, 2207–2217. doi: 10.1038/ismej.2014.77, PMID: 24858782 PMC4992085

[ref78] Walke J. B. Becker M. H. Loftus S. C. House L. L. Teotonio T. L. Minbiole K. P. C.. (2015). Community structure and function of amphibian skin microbes: an experiment with bullfrogs exposed to a chytrid fungus. PLoS One 10:e0139848. doi: 10.1371/journal.pone.0139848, PMID: 26445500 PMC4596541

[ref79] West A. G. Waite D. W. Deines P. Bourne D. G. Digby A. McKenzie V. J.. (2019). The microbiome in threatened species conservation. Biol. Conserv. 229, 85–98. doi: 10.1016/j.biocon.2018.11.016

[ref80] Wickham H. (2016). ggplot2: elegant graphics for data analysis. R package version 3.3.5. Available at: https://ggplot2.tidyverse.org

[ref81] Woodhams D. C. Alford R. A. Antwis R. E. Archer H. Becker M. H. Belden L. K.. (2015). Antifungal isolates database of amphibian skin-associated bacteria and function against emerging fungal pathogens. Ecology 96:595. doi: 10.1890/14-1837.1

[ref82] Woodhams D. C. Bletz M. Kueneman J. McKenzie V. (2016). Managing amphibian disease with skin microbiota. Trends Microbiol. 24, 161–164. doi: 10.1016/j.tim.2015.12.010, PMID: 26916805

[ref83] Woodhams D. C. LaBumbard B. C. Barnhart K. L. Becker M. H. Bletz M. C. Escobar L. A.. (2018). Prodigiosin, violacein, and volatile organic compounds produced by widespread cutaneous bacteria of amphibians can inhibit two *Batrachochytrium* fungal pathogens. Microb. Ecol. 75, 1049–1062. doi: 10.1007/s00248-017-1095-7, PMID: 29119317

[ref84] Zhang P. Papenfuss T. J. Wake M. H. Qu L. Wake D. B. (2008). Phylogeny and biogeography of the family Salamandridae (Amphibia: Caudata) inferred from complete mitochondrial genomes. Mol. Phylogenet. Evol. 49, 586–597. doi: 10.1016/j.ympev.2008.08.020, PMID: 18801447

