# Supplementary material for: Strong restructuration of skin microbiota during captivity challenges ex-situ conservation of amphibians
Source: Front Microbiol. 2023 Feb 20;14:1111018. doi: 10.3389/fmicb.2023.1111018 (PMC9986596; doi:10.3389/fmicb.2023.1111018)
Supplement: Supplementary file 1 [file Data_Sheet_1.PDF]

Supplementary Material

Strong restructuration of skin microbiota during captivity challenges *ex-situ* conservation of amphibians.

This PDF includes:

Supplementary Figures 1 to 4

A separate file (Supplementary_information_tables.xslx) includes:

Supplementary Tables 1 to 12

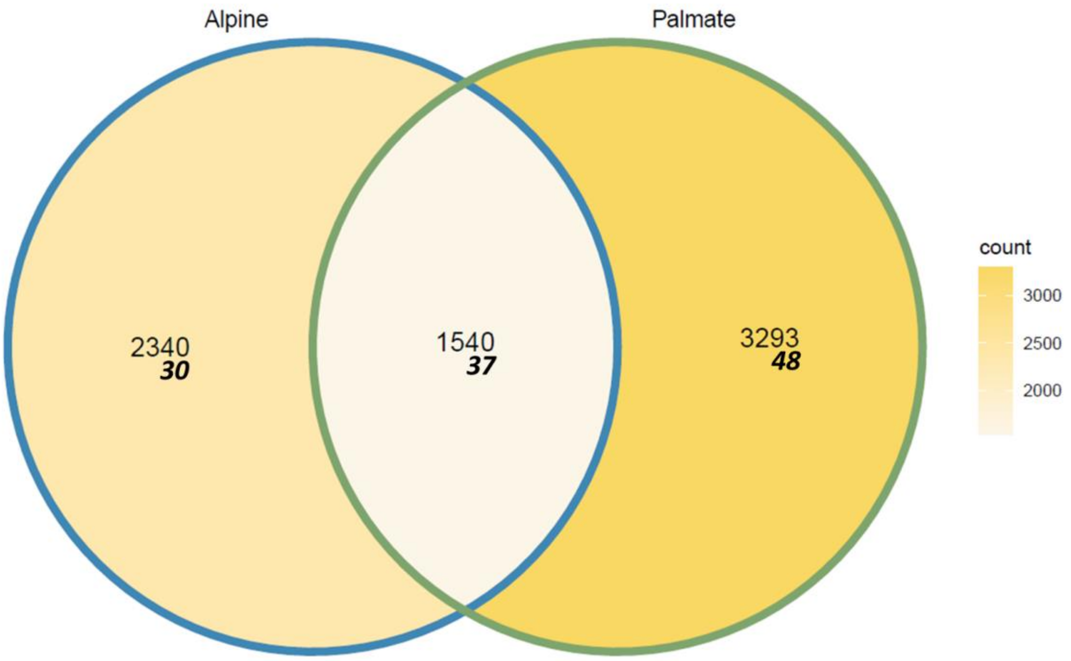

Supplementary Figure 1. Venn diagrams of the total number of phylotypes shared between wild alpine and palmate newts. The intensity of the yellow color is proportional to the sum of ASVs in each group. The number of *Bd*-inhibitory phylotypes among the total ASVs is indicated in bolded italics.

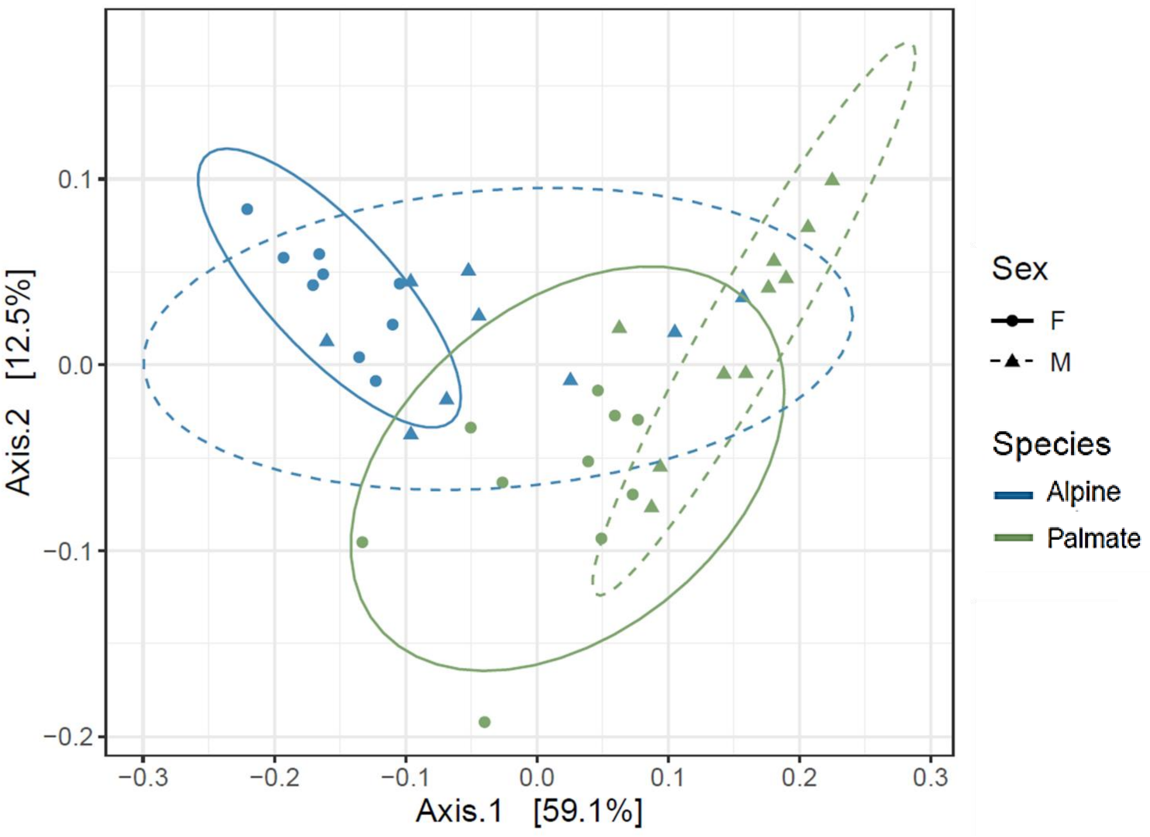

Supplementary Figure 2. PCoA representing the beta diversity (calculated as the weighted Unifrac distance) of the skin microbiota of wild newts. Each dot represents the microbiota of a single individual. The species of each newt is indicated by the color of its datapoint, and its sex (F, female; M, male) by the shape of the datapoint as well as the line-type of the ellipse.

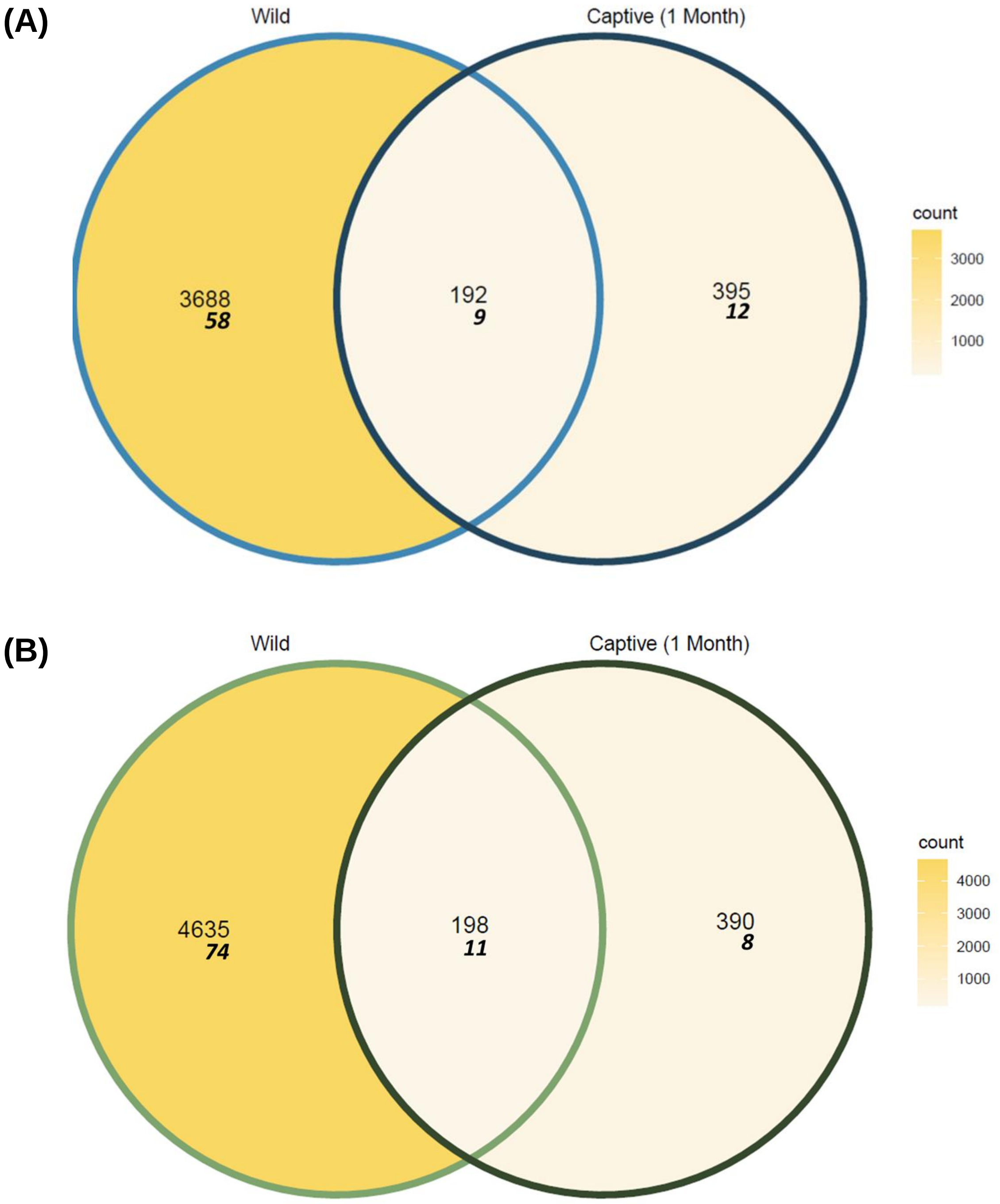

Supplementary Figure 3. Venn diagrams of the total number of phylotypes shared between wild and captive (after one month in captivity) alpine (A) and palmate (B) newts. The intensity of the yellow color is proportional to the sum of ASVs in each group. The number of *Bd*-inhibitory phylotypes among the total ASVs is indicated in bolded italics.

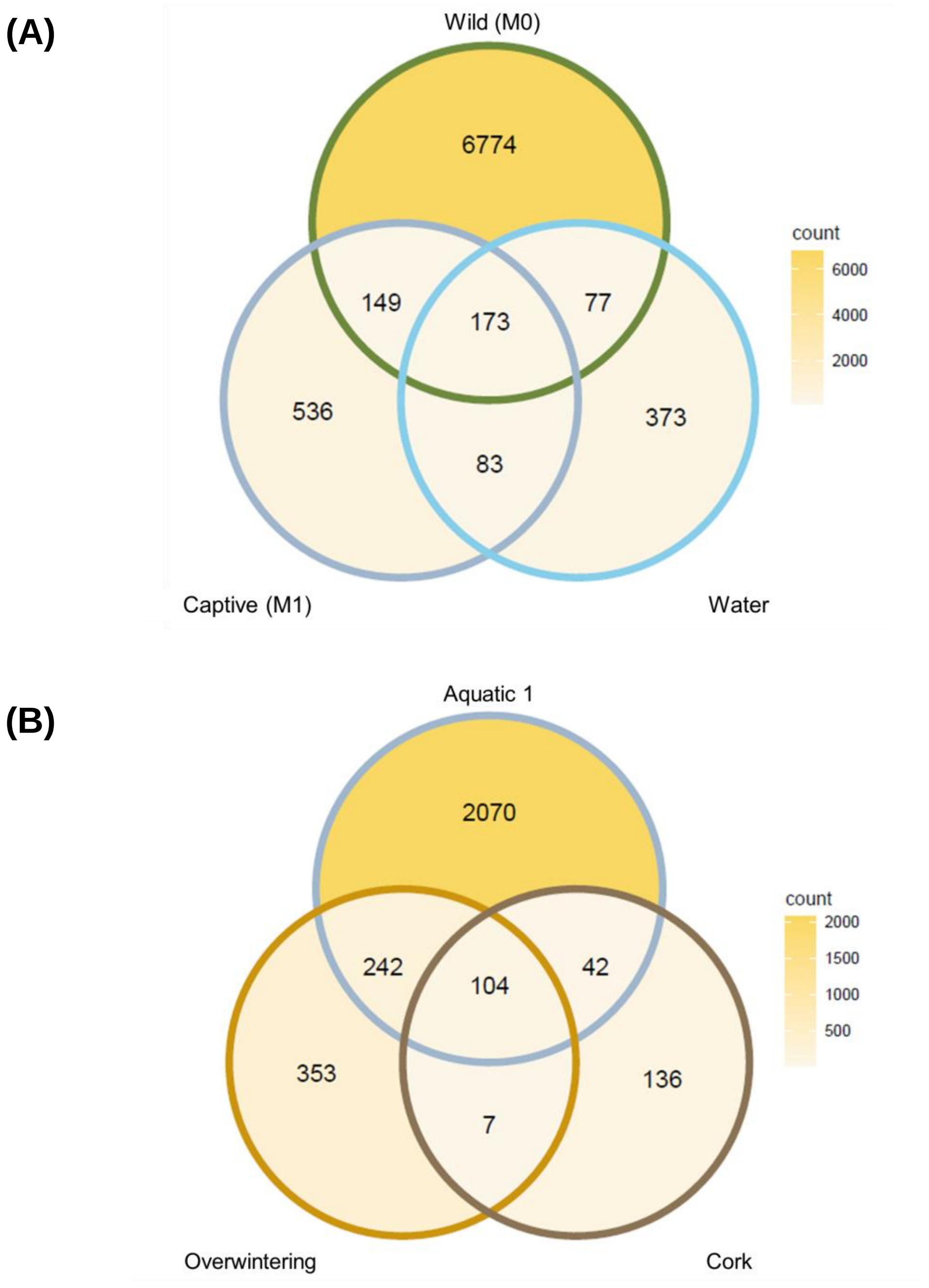

Supplementary Figure 4. Venn diagrams of the total number of phylotypes in the skin microbiota of captive newts that may have been acquired through contamination by the water in the aquaria (A) or by the cork in the overwintering containers (B). Eighty-three of the new ASVs that colonized the newts' microbiota when they were placed in captivity (Captive) may have come from the water in the aquaria (Water). Seven on the ASVs acquired during the overwintering may have come from the cork. The intensity of the yellow color is proportional to the sum of ASVs in each group.

A separate file (Supplementary_information_tables.xslx) includes:

Supplementary Table 1. Significant log2 fold difference in abundance of bacterial phylotypes in the skin microbiota of wild newts. Each line describes a phylotype differentially abundant in the microbiota of wild palmate newts compared to wild alpine newts. *Bd*-inhibitory taxa are indicated by a "+" symbol (Bd-inhibition). The direction of the change in abundance is provided with its amplitude (Log2 fold change) and its standard error (SE). Blank spaces indicate unknown phylogenetic attribution according to SILVA.

Supplementary Table 2. Significant log2 fold changes in abundance of bacterial phylotypes in the skin microbiota of alpine newts over a short-term transfer from the wild to captivity. Each line describes a phylotype differentially abundant in the microbiota of captive newts (after one month in captivity) compared to wild newts. Phylotypes that were specifically differentially abundant in the microbiota of alpine newts (unique; n = 57) and that which also differed in abundance in palmate newts (common; n = 138) are presented separately (Variation specificity). *Bd*-inhibitory taxa are indicated by a "+" symbol (Bd-inhibition). The direction of the change in abundance is provided with its amplitude (Log2 fold change) and its standard error (SE). Blank spaces indicate unknown phylogenetic attribution according to SILVA.

Supplementary Table 3. Significant log2 fold changes in abundance of bacterial phylotypes in the skin microbiota of palmate newts over a short-term transfer from the wild to captivity. Each line describes a phylotype differentially abundant in the microbiota of captive newts (after one month in captivity) compared to wild newts. Phylotypes that were specifically differentially abundant in the microbiota of palmate newts (unique; n = 129) and that which also differed in abundance in alpine newts (common; n = 138) are presented separately (Variation specificity). *Bd*-inhibitory taxa are indicated by a "+" symbol (Bd-inhibition). The direction of the change in abundance is provided with its amplitude (Log2 fold change) and its standard error (SE). Blank spaces indicate unknown phylogenetic attribution according to SILVA.

Supplementary Table 4. Pairwise contrasts in microbiota richness (Chao1) between phases, using estimated marginal means. Both alpine and palmate newt species were considered together here, as our models showed that their microbiota richness was similarly affected by phase shifts. The contrasts indicate which phases were compared, using lexicon from Table 1.

Supplementary Table 5. Pairwise contrasts in microbiota diversity (Shannon) between phases, using estimated marginal means. Alpine and palmate newts are presented separately (Species) as our models showed that their microbiota diversity was differentially affected by phase shifts. The contrasts indicate which phases were compared, using lexicon from Table 1.

Supplementary Table 6. Pairwise contrasts in beta diversity (calculated as the weighted Unifrac distance) of the microbiota of alpine and palmate newts between phases, using pairwise PERMANOVA. Alpine and palmate newts are presented separately (Species) as our models showed that their microbiota community structure was differentially affected by phase shifts. The contrasts indicate which phases were compared, using lexicon from Table 1.

Supplementary Table 7. Significant log2 fold changes in abundance of bacterial phylotypes in the skin microbiota of captive alpine newts over the phase-shift from the first aquatic phase to the overwintering phase. Each line describes a phylotype differentially abundant in the microbiota of overwintering newts compared to newts in the first aquatic phase. Phylotypes that were specifically differentially abundant in the microbiota of alpine newts (unique; n = 66) and that which also differed in abundance in palmate newts (common; n = 127) are presented separately (Variation specificity). *Bd*-inhibitory taxa are indicated by a "+" symbol (Bd-inhibition). The direction of the change in abundance is provided with its amplitude (Log2 fold

change) and its standard error (SE). Blank spaces indicate unknown phylogenetic attribution according to SILVA.

Supplementary Table 8. Significant log2 fold changes in abundance of bacterial phylotypes in the skin microbiota of captive palmate newts over the phase-shift from the first aquatic phase to the overwintering phase. Each line describes a phylotype differentially abundant in the microbiota of overwintering newts compared to newts in the first aquatic phase. Phylotypes that were specifically differentially abundant in the microbiota of palmate newts (unique; *n* = 64) and that which also differed in abundance in alpine newts (common; *n* = 127) are presented separately (Variation specificity). *Bd*-inhibitory taxa are indicated by a "+" symbol (Bd-inhibition). The direction of the change in abundance is provided with its amplitude (Log2 fold change) and its standard error (SE). Blank spaces indicate unknown phylogenetic attribution according to SILVA.

Supplementary Table 9. Significant log2 fold changes in abundance of bacterial phylotypes in the skin microbiota of captive alpine newts over the phase-shift from the overwintering period to the second aquatic phase. Each line describes a phylotype differentially abundant in the microbiota of aquatic newts compared to overwintering newts. Phylotypes that were specifically differentially abundant in the microbiota of alpine newts (unique; *n* = 47) and that which also differed in abundance in palmate newts (common; *n* = 97) are presented separately (Variation specificity). *Bd*-inhibitory taxa are indicated by a "+" symbol (Bd-inhibition). The direction of the change in abundance is provided with its amplitude (Log2 fold change) and its standard error (SE). Blank spaces indicate unknown phylogenetic attribution according to SILVA.

Supplementary Table 10. Significant log2 fold changes in abundance of bacterial phylotypes in the skin microbiota of captive palmate newts over the phase-shift from the overwintering period to the second aquatic phase. Each line describes a phylotype differentially abundant in the microbiota of aquatic newts compared to overwintering newts. Phylotypes that were specifically differentially abundant in the microbiota of palmate newts (unique; *n* = 59) and that which also differed in abundance in alpine newts (common; *n* = 97) are presented separately (Variation specificity). *Bd*-inhibitory taxa are indicated by a "+" symbol (Bd-inhibition). The direction of the change in abundance is provided with its amplitude (Log2 fold change) and its standard error (SE). Blank spaces indicate unknown phylogenetic attribution according to SILVA.

Supplementary Table 11. Significant log2 fold changes in abundance of bacterial phylotypes in the skin microbiota of captive alpine newts over 10 months in captivity. Each line describes a phylotype differentially abundant in the microbiota of newts in their 10th month in captivity compared to initial abundance upon collection in the wild. Phylotypes that were specifically differentially abundant in the microbiota of alpine newts (unique; *n* = 46) and that which also differed in abundance in palmate newts (common; *n* = 143) are presented separately (Variation specificity). *Bd*-inhibitory taxa are indicated by a "+" symbol (Bd-inhibition). The direction of the change in abundance is provided with its amplitude (Log2 fold change) and its standard error (SE). Blank spaces indicate unknown phylogenetic attribution according to SILVA.

Supplementary Table 12. Significant log2 fold changes in abundance of bacterial phylotypes in the skin microbiota of captive palmate newts over 10 months in captivity. Each line describes a phylotype differentially abundant in the microbiota of newts in their 10th month in captivity compared to initial abundance upon collection in the wild. Phylotypes that were specifically differentially abundant in the microbiota of palmate newts (unique; *n* = 126) and that which also differed in abundance in alpine newts (common; *n* = 143) are presented separately (Variation specificity). *Bd*-inhibitory taxa are indicated by a "+" symbol (Bd-inhibition). The direction of the change in abundance is provided with its amplitude (Log2 fold change) and its

standard error (SE). Blank spaces indicate unknown phylogenetic attribution according to SILVA.
